# Single-centre experience and practical considerations of the benefit of a second cochlear implant in bilaterally deaf adults

**DOI:** 10.1007/s00405-020-06315-x

**Published:** 2020-09-05

**Authors:** Dominik Péus, Andreas Pfluger, Sophia Marie Häussler, Steffen Knopke, Manuel Christoph Ketterer, Agnieszka J. Szczepek, Stefan Gräbel, Heidi Olze

**Affiliations:** 1 Department of Otorhinolaryngology, Campus Virchow Klinikum, Charité-Universitätsmedizin Berlin, Augustenburger Platz 1, 13353 Berlin, Germany; 2 Department of Otorhinolaryngology, Kantonsspital Baselland, Liestal, Switzerland

**Keywords:** Cochlear implant, CI, Bilaterally deafened, Hearing loss, Quality of life, Speech recognition

## Abstract

**Purpose:**

Bilateral cochlear implant (CI) implantation is increasingly used in the auditory rehabilitation of bilaterally deafened adults. However, after successful unilateral implantation, objective patient counselling is essential.

**Methods:**

We investigated the extra benefit of a second CI in adults in terms of health-related quality of life, tinnitus, stress, anxiety, depression, quality of hearing, and speech recognition. Hearing ability was assessed by using the Freiburg monosyllable speech discrimination test (FB MS) and the Oldenburg sentence test with azimuth variations. In a prospective patient cohort, we administered validated questionnaires before a CI, after a first CI and after a second CI implantation.

**Results:**

The study included 29 patients, made up of nine women and 20 men. The median time between the first and the second implantation was 23 months. The mean total NCIQ score and TQ before a CI improved significantly after both implantations. Stress, anxiety, and depression were stable over time and were not significantly affected by CI implantations. Speech recognition with noise significantly improved after the first and again after the second CI. Correlation analysis showed a strong connection between auditory performance and HRQoL.

**Conclusion:**

We demonstrated that a unilateral CI benefitted many fields and that the second sequential CI leads again to additional improvement. Bilateral CI implantation should, therefore, be the standard form of auditory rehabilitation in deafened adults.

## Introduction

The number of bilaterally deafened adults is increasing, mainly due to a constantly ageing society. A bilateral CI is a widely accepted medical practice in infants and children h [1]. However, in post-lingual bilaterally deafened adults, there is ongoing discussion regarding outcomes as well as medical and surgical safety concerns [2]. It is known that CIs allow people with hearing disorders to regain auditory perception and an acoustic understanding of speech. Among the positive consequences of a bilateral CI versus a unilateral CI are improved sound localisation, enhanced speech perception, and a reduced risk of being "off-air" [3–5]. The theoretically potential disadvantages include bilateral vestibular alterations, surgical complications, and cost [6–8]. The debate about cost-effectiveness is particularly controversial [9–11]. Up to this point, most research investigating bilateral CI supply has focused on auditory improvement, including sound localisation and enhanced speech perception, but little is known about the subjectively experienced changes created by a CI, and very few studies have analysed the health-specific quality of life changes due to a second CI [12, 13].

The list of indications for cochlear implantation has expanded in the last decade. As a result, increased numbers of adult post-lingually deafened patients are scheduled for a second CI. To measure the impact of CIs on patients' lives, our study group uses a test battery, which in addition to the auditory performance, assesses the subjective perception of stress factors, patients' resources, ways of coping with stress, and the degree of anxiety and depressive symptoms [14]. In the past, the benefit of the second CI was investigated in a retrospective manner, which had some limitations, because the patients had to reply to the questionnaires retrospectively, and the period between implantation and questionnaire replies was quite heterogeneous. In the meantime, the test battery was refined and is routinely used to assess the patients with asymmetric hearing loss, after a positive impact of the first CI. The present study was designed as a prospective cohort study and aimed to investigate the impact of a second CI on the auditory performance, perceived stress, patients' resources, coping, and the degree of anxiety and depressive symptoms of implanted subjects.

## Methods

### Patients

Between July 2010 and May 2016, 29 bilaterally deaf adult patients who were scheduled for a bilateral CI implantation were included in this study. The implantations were performed sequentially, and the median time between the implantations was 23 months. Patients were interviewed before the first implantation and six months after the first and the second implantation.

The cohort consisted of nine women and 20 men, averaging 57.63 years of age. The period of deafness was determined based on the reported time when a patient felt that wearing a hearing aid had stopped being of significant benefit, and averaged 18.92 years. The mean value Freiburg monosyllable speech discrimination test (FB MS) for implanted ears was 6.05 (SD = 11.85), while on contralateral ears, it was 10.87 (SD = 16.42).

### Variables and goals

For each patient, we collected data at three time points: prior to the first CI, six months after the first implantation, and again six months after second implantation. Patients filled paper questionnaires, as described by Bruggemann et al. (2017), including the Nijmegen Cochlear Implantation Questionnaire (NCIQ), the Tinnitus Questionnaire (TQ), the Perceived Stress Questionnaire (PSQ), the brief German COPE (COPE), the General Depression Scale (ADS), and the Generalised Anxiety Disorder (GAD) [15–22]. Subjective hearing ability was assessed using the Oldenburg Inventory, and audiometric measurements were performed in the audiometry unit of the otolaryngology department.

Speech perception was measured in all patients before the CI, using the FB MS. This involved presenting patients with 2 × 20 monosyllabic words at a volume of 65 dB HL in quiet conditions. After the first and second CIs, the FB MS and Oldenburger Satz test (OLSA) were used [21]. The OLSA consists of a five-word sentence used to assess speech perception in noisy conditions and is defined as a signal-to-noise ratio in dB SPL (SNR). The OLSA was used in the following five test conditions: after the first CI, (1) speech and noise were presented from the front, (2) noise was directed at the implanted ear and speech was directed at the deaf ear and (3) vice versa. After a second CI, (4) speech and noise were presented from the front and (5) speech was directed at the first implanted ear, while noise was directed at the second implanted ear. Azimuth variation for speech and noise presentation simulates an everyday hearing situation.

The primary objective of this prospective analysis was to identify predictive values for a successful second CI, defined by an increase in health-related quality of life and better performance in the monosyllable test and the more difficult speech perception tests.

### Statistical procedure

Twenty-nine patients were included in this study. Statistical evaluation was carried out using the software program SPSS (IBM Corp. Released 2017. IBM SPSS Statistics for Windows, Version 25, Armonk, NY: IBM Corp.). Frequency tables, mean values, and standard deviations were calculated descriptively. The Kolmogorov–Smirnov test was used to test for distribution normality, and the Wilcoxon test was used to test the significance of differences before and following implantation. Although some quantitative data were available, there were insufficient data for regression analysis, and so we report effect sizes herein, using Pearson's correlation test. Significance was set at *p* < 0.05.

## Results

### Additional health-related quality of life

**Fig. 1:**
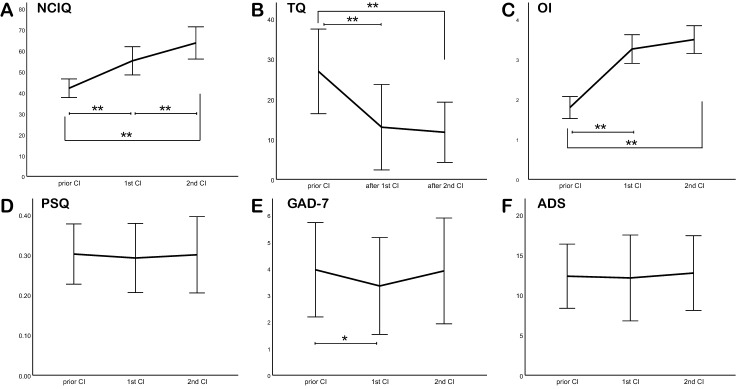
Changes in mean total scores for NCIQ, TQ, OI, PSQ, GAD-7, and ADS prior to a CI, after a first CI and after a second CI. Changes to the NCIQ, TQ, OI, and PSQ subscales behaved similarly to the mean total scores and were therefore not plotted. Error bars: 95% confidence interval. *p < 0.05; **p < 0.01; ***p < 0.001

The disease-specific quality of life measured by the NCIQ improved significantly after the first CI and again after the second CI. The mean total NCIQ score before any CI was 42.80 ± 17.84. After a first CI, it improved to 55.66 ± 16.06 (*p* < 0.000) and then improved further to 63.28 ± 18.53 (*p* = 0.012) following the second CI (see Fig. 1a). NCIQ subdomains, including basic sound perception, advanced sound perception, speech production, self-esteem, and social interactions, significantly improved after both CIs, except for subdomain NCIQ 3, i.e. speech production (see Table 1).

**Table 1:** Test scores prior to CI, after a first CI, and after a second CI

	Mean prior to CI	SD	Mean after 1st CI	SD	Mean after 2nd CI	SD	*Z* (Comparison between. prior and first)	*p*	*Z* (Comparison between. first and second)	*p*
FM MS (65 dB SPL)	6.05	11.85	48.69	28.94	66.59	19.11	− 3.041	**0.002**	− 3.411	**0.001**
NCIQ 1	38.57	18.45	56.21	24.11	70.94	22.84	− 3.363	**0.001**	− 3.924	**0.000**
NCIQ 2	35.03	15.89	53.86	20.55	61.18	24.49	− 3.990	**0.000**	− 3.576	**0.000**
NCIQ 3	60.49	17.60	69.66	17.69	68.48	18.09	− 2.572	**0.010**	− 2.373	**0.018**
NCIQ 4	44.60	16.37	51.16	15.75	60.82	18.00	− 2.681	**0.007**	− 3.118	**0.002**
NCIQ 5	38.69	15.10	50.09	19.65	56.32	23.90	− 3.196	**0.001**	− 3.036	**0.002**
NCIQ 6	40.05	17.84	53.96	20.69	65.39	20.40	− 3.484	**0.000**	− 3.685	**0.000**
NCIQ total	42.80	11.34	55.66	16.08	63.28	18.53	− 3.968	**0.000**	− 3.711	**0.000**
TQ emotional distress	6.48	4.75	3.76	4.94	2.31	3.24	− 2.196	**0.028**	− 1.899	0.058
TQ cognitive distress	4.95	3.84	3.24	4.37	2.25	3.34	− 1.969	**0.049**	− 2.223	**0.026**
TQ intrusiveness	7.19	4.74	4.38	4.94	3.00	3.79	− 2.728	**0.006**	− 2.182	**0.029**
TQ auditory perceptual difficulties	6.00	4.70	3.19	4.49	2.63	3.30	− 3.051	**0.002**	− 1.964	0.050
TQ sleep disturbance	2.24	2.53	0.90	2.14	0.63	1.02	− 2.614	**0.009**	− 2.680	**0.007**
TQ somatic complaints	1.10	1.37	0.71	1.23	0.19	0.40	− 1.642	0.101	− 1.973	**0.049**
TQ total	27.52	19.67	15.68	20.37	11.00	13.44	− 2.877	**0.004**	− 2.047	**0.041**
PSQ worries	0.26	0.24	0.22	0.23	0.23	0.28	− 1.440	0.150	− 1.326	0.185
PSQ tension	0.29	0.20	0.32	0.22	0.30	0.26	− 0.325	0.746	− 0.469	0.639
PSQ joy	0.58	0.22	0.56	0.25	0.58	0.29	− 0.801	0.423	− 0.162	0.871
PSQ demands	0.30	0.18	0.25	0.24	0.24	0.23	− 2.086	**0.037**	− 1.083	0.279
PSQ total	0.31	0.17	0.30	0.19	0.23	0.28	− 0.681	0.496	− 0.259	0.796
COPE avoidance	9.85	2.24	8.96	2.46	9.96	2.53	− 1.490	0.136	− 0.105	0.917
COPE seeking support	13.04	3.61	11.77	3.49	9.87	3.24	− 0.979	0.327	− 3.012	**0.003**
COPE positive thinking	14.42	3.61	14.23	3.90	11.04	3.47	− 0.033	0.974	− 3.063	**0.002**
COPE active problem solving	11.04	3.47	9.38	2.30	8.96	3.14	− 1.691	0.091	− 2.229	**0.026**
Depression (ADS)	13.28	8.84	12.45	11.50	13.05	10.93	− 0.604	0.546	− 0.174	0.862
GAD-7 anxiety	3.75	4.56	3.29	3.94	4.10	3.90	− 1.973	**0.049**	− 0.244	0.807
OI quiet setting	2.06	0.73	3.67	0.85	4.00	0.80	− 4.521	**0.000**	− 4.203	**0.000**
OI noise interference	1.47	0.48	2.94	0.94	2.95	0.90	− 4.313	**0.000**	− 4.112	**0.000**
OI directional listening	2.00	0.78	2.80	1.06	3.37	0.89	− 3.345	**0.001**	− 3.950	**0.000**
OI total	1.81	0.55	3.22	0.87	3.46	0.78	− 4.445	**0.000**	− 4.198	**0.000**

Significance was set at *p* < 0.05 of bold highlighting

### Audiological improvement—unilateral versus bilateral CI

The mean value FB MS for the first implanted ears was 6.05 ± 11.85; on contralateral ears, it was 10.87 ± 16.42. After first CI, FB MS increased to 48.69 ± 28.94 (*p* < 0.001). After the second CI, the FB MS improved further to 66.43 ± 19.57 (*p* = 0.002) with bilaterally active CI. After the second CI operation, both ears were also investigated with only one active CI. The second operated ear performed slightly better 56.59 ± 19.24 versus the first operated ear 50.68 ± 22.16. However, the difference was not statistically significant.

When speech and noise were presented from the front, the mean OLSA SNR was 0.368 ± 3.109 dB SPL with a bilateral CI, while the mean OLSA SNR was 1.881 ± 4.459 dB SPL with a unilateral CI. The 1.513 dB SPL difference was significant (*p* = 0.002). If the speech was presented to the first implanted ear, and noise to the deaf ear, the mean OLSA SNR was − 4.241 ± 5.338 dB respective to the contralateral CI − 3.518 ± 5.00 dB SPL. The difference, in this case, was not significant (*p* = 0.115). Speech understanding measures were justifiably the lowest observed when noise was presented to the CI ear and speech to the deaf ear at 7.231 ± 4.65 dB SPL in unilaterally implanted patients.

### Subjective hearing ability

The first CI resulted in a significant improvement in the mean total OI score. Before a first CI, the mean total OI score was 1.81 ± 0.55, but this improved to 3.22 ± 0.55 after the first CI. All three subdomains, "OI quiet", "OI noise interference", and "OI directional listening", significantly improved, and further progress after a second CI was observed in all OI subdomains.

### Tinnitus annoyance

In the study cohort, tinnitus annoyance was reduced by the first CI to a low level and declined even further after secondary surgery. Twenty-one patients (72%) reported tinnitus of some kind before CI implantation, with a mean total TQ score of 27.52 ± 19.67. TQ decreased after the first CI to 15.68 ± 20.37 (*p* = 0.004) and decreased further after the second to 11.00 ± 13.44 (*p* = 0.041). Before any CI, five patients (23.8%) reported decompensated tinnitus (total TQ score > 46). After the first CI, two patients in the decompensated tinnitus subgroup achieved intermediate levels (total TQ score 31–46). One patient reported a complete absence of tinnitus, and two reported continuous high tinnitus annoyance. These two patients profited from the second CI, with one achieving low-level and the other intermediate-level tinnitus. Each of the three patients (14%) with intermediate tinnitus annoyance before the first CI reported a decrease to low tinnitus levels (total TQ score 0–30) after the procedure, and this reduction persisted after the second CI. All subscales scores, including emotional and cognitive distress, intrusiveness, auditory perceptual difficulties, sleeping disturbances, but somatic complaints*,* declined significantly after the first CI and again after the second CI (see Table 1).

### Psychosocial burden

To measure the psychosocial burden of CI patients, PSQ, COPE, GAD 7, and ADS questionnaires were filled by the patients and analysed by qualified staff. The perceived stress questionnaire showed relatively low levels of preoperative stress, with a mean total PSQ of 0.31 ± 0.17. PSQ total scores remained stable over time. Only the PSQ subscale demands demonstrated a significant reduction after the first CI (*p* = 0.037), and this value remained stable after the second procedure (see Fig. 1d), thereby implying that neither surgery increased patients' stress.

The COPE questionnaire has four subdomains, namely avoidance, seeking support, positive thinking, and active problem-solving. COPE scores have not changed significantly after the first CI. However, significant reductions in three of the four subdomains (avoidance, seeking support, and positive thinking) were observed after the second implantation (see Table 1). That may be because coping and problem-solving strategies are less essential after a second CI, thus removing some of the limitations of monaural hearing.

The mean score of the ADS depression questionnaire was 13.28 ± 8.84 before any CI, and 10 of the 29 (34.4%) patients scored 16 or more, which is a degree of depression that is understood to be deserving clinical treatment. There was a slight reduction after the first CI, and a very slight increase after second CI. However, these changes were not significant (*p* = 0.546 and *p* = 0.862).

The mean of the GAD-7 questionnaire, which assesses fear, was 3.75 ± 4.56 pre-CI, which indicates minimal to mild anxiety. After the first CI, the mean GAD-7 declined to 3.29 ± 3.94 (*p* = 0.049). After the second CI, it was 4.10 ± 3.90 (*p* = 0.807). Before CI, nine of the 29 (31%) patients reported at least mild anxiety (4–9 pt.) [20], and this has not changed after either CI.

### Correlation of health-related quality of life, psychosocial burden, and hearing

**Fig. 2:**
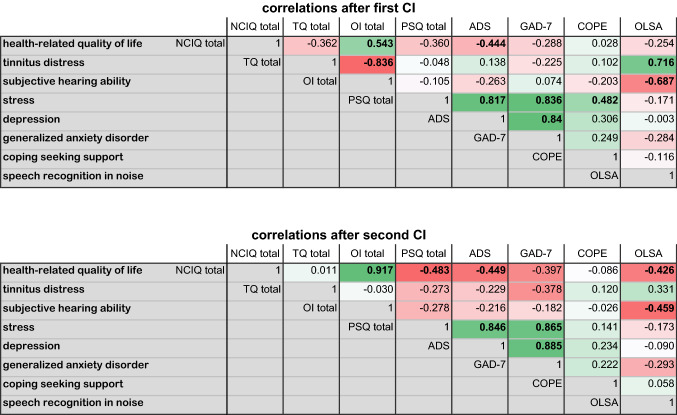
Correlation heat map between health-related quality of life, psychosocial burden, subjective quality of hearing, and speech recognition. Pearson correlation coefficients after the first and second CIs. Significant correlations are highlighted in bold (*p* < 0.05). Green boxes refer to positive correlations. Red boxes refer to negative correlations

Pearson correlations were performed in order to gain a better understanding of the relations between HRQoL (NCIQ), tinnitus distress (TQ), stress (PSQ), generalised anxiety disorder (GAD-7), depression (ADS), coping (COPE), subjective quality of hearing (OI), and hearing performance with background noise. The heat maps summarise the correlations after the first and after the second CI (see Fig. 2). For the sake of clarity, we used two mean total scores for NCIQ, TQ, PSQ, GAD-7, and ADS, namely the COPE subscale "seeking support" and the signal-to-noise ratio presented from the front. The first main finding is that after the first CI, tinnitus distress correlated significantly with subjective hearing ability and speech recognition, and after the second CI, tinnitus distress significantly decreased and the previous correlation disappeared. The second main finding is that the correlation between HRQoL and subjective hearing ability is quite strong (Pearson correlation coefficient = 0.543) and becomes even more significant after the second CI (Pearson correlation coefficient = 0.917), highlighting the importance of sufficient hearing capacity for a good quality of life.

## Discussion

In the present work, we used a prospective study to follow 29 post-lingually bilaterally deaf patients from the moment when they were scheduled for the first cochlear implantation, up to six months after the second cochlear implantation. At present, there is an ongoing global discussion about bilateral cochlear implantation as a standard procedure for bilaterally deafened people. Apart from medical and audiological issues, this discussion involves health politics. Here, we provide evidence about the medical, psychological, and social benefits in patients who were bilaterally, subsequently implanted with CI.

One of our main findings was that HRQoL significantly improved, not only after the first CI implantation but also after the second CI. This remarkable additional improvement was seen in all subdomains of the NCIQ: primary sound perception, advanced sound perception, speech production, self-esteem, activity, and social interactions. Our results corroborate these of others [10, 12, 23] and fully justify the medical effort.

In addition to the quality of life, speech comprehension, and directional hearing improved after first and after second CI implantation, in agreement with already published data [3]. Also, the quality of life and subjective hearing ability strongly correlated. All three subscales and the mean total score for subjective hearing ability OI (quiet setting, noise interference, and directional listening) improved mainly after the first and to a lesser extent after the second CI (Fig. 1c)—in contrast to NCIQ (Fig. 1a), where a steeper graphical incline was observed. HRQoL strongly correlated with subjective hearing ability, whereas it did not correlate with SNR performance after the first CI, but it did so after the second CI. Therefore, to assess the psychosocial changes caused by a second CI, questionnaires are a useful augmentation to standard speech perception tests and subjective hearing assessment.

Many studies report improved speech perception—and notably, directional listening—after a second CI [12, 24]. The OI subdomain of directional listening improved relative to the other two subdomains, most of all following the second CI in our study, which seems logical, because two functional hearing organs are required for the downstream central auditory pathways to achieve the spatial localisation of sound sources [25]. Interestingly, average head shadow effect in the monaural condition was similar to a bilaterally active CI, which might be explained by temporal and spectral differences of spatially different speech and noise sources (the squelch effect), but electronic noise filters and different CI cues may also contribute in this regard [26]. In the unilateral CI situation, with noise presented to the CI ear and speech to the deaf ear, the mean SNR was 7.231 ± 4.65 dB SPL. An SNR value of 7.231 dB SPL (different to a bilateral CI by 10.749 dB SPL) indicates inferior speech recognition. However, the quantitative audiometric measurements correlated only moderately with statistical significance in the binaural situation but correlated better with subjective hearing ability.

Evidence accumulates about the reduction in tinnitus annoyance being closely related to the improvement in HRQoL [27–29]. In the present study, although the patients with decompensated tinnitus benefitted in particular from a second CI, there was no statistically significant correlation between NCIQ and TQ. In the previous data published by Knopke et al. (2017), tinnitus was an inclusion criterion, and there was a higher proportion of patients with decompensated tinnitus. In the present study, the study sample was relatively small, and the patients had relatively low tinnitus annoyance, which might explain our results. However, there was a strong negative correlation between tinnitus distress and subjective hearing ability. In summary, tinnitus reduction after second cochlear implantation does not account for the improvement in the quality of life.

We also demonstrated that the subjective ability of hearing correlates positively with the HRQoL and negatively with elevated scores in the depression assessment (see also Fig. 2). This supports the importance of hearing ability in patients' subjectively experienced HRQoL. Although the information about a type of specific treatment for depression was not assessed, cochlear implantation likely enabled the patients to attend conversational psychotherapeutic therapy.

Before CI, nine of 29 (31%) patients reported at least mildly severe anxiety [20], which negatively correlated with the quality of hearing and support-seeking. As the hearing performance improved, this correlation became insignificant.

A great deal of effort has been spent on identifying the predictive values for the outcome of a second CI. In summary, no feature of an individual can predict the outcome—even advanced age, a long duration of deafness, or a long interval between implantations are not significant negative predictors [30]. However, all patients prefer to implant the poorer ear first (in many cases, the ear that was suppressed over many years), and they wait until the second ear worsens*.* This fact produces an analytical heterogeneity. Additionally, the current study could not find predictive values by using regression analysis. The improvement in the HRQoL after the second CI might also be explained as a result of the better ear and upstream connections being suppressed over a shorter period, and therefore hearing performance and experience after the second CI being judged by CI recipients as very satisfactory.

## Conclusion

The present study demonstrates that in bilaterally deafened adult patients, a second CI implantation improves HRQoL, tinnitus annoyance, speech comprehension under noisy conditions, and subjective quality of hearing compared to a one-sided CI condition. The extent of improvement in auditory performance was positively correlated with the improved health-related quality of life. No effect of implantation on depression, stress, and anxiety disorders was observed.

There are no measures currently available after fitting the first CI that help predict whether a patient is a suitable candidate for a second CI; however, nearly all patients profit from a second CI in several respects. Specialists should thus actively talk with patients about a second CI in unilateral CI recipients to inform them objectively about the risks and possible benefits of the second implantation. A bilateral CI implantation should be encouraged in most cases as a standard form of auditory rehabilitation in deafened children and adults—with very few exceptions.
